# A Manual of Clinical Medicine, and Physical Diagnosis

**Published:** 1855-11

**Authors:** 


ART. XII.—*A Manual of Clinical Medicine, and Physical Diagnosis.* By T. H. TANNER, M. D., Licentiate of the Royal College of Physicians; Physician to the Hospital for Women, etc. To which is added the Code of Ethics of the American Medical Association. Philadelphia: Blanchard & Lea. 1855.

This is a reprint of a clever little duodecimo, after the fashion of "What to observe at the Bedside." As tending to promote system and care in the examination of patients, it is deserving of encouragement, and we hope it may have a large sale.

No person feels the necessity of some system of clinical examinations and records, more than the editor of a medical periodical. Cases, in themselves deeply interesting, are often reported for his pages with facts of the utmost importance to its appreciation omitted. Vague and indescriptive terms are used in such a manner as to convey no definite pathological idea. These faults arise from a habit of careless clinical observation, and from the want of that order and system which belong *only* to him who records his cases. Such books as these, if largely distributed among the profession, would go far to correct this serious evil.

